# In this month’s Bulletin

**DOI:** 10.2471/BLT.17.000617

**Published:** 2017-06-01

Margaret E Kruk et al. (390) introduce this special issue on why and how to measure the quality of health-care provision.

In the news section**, **Amy Guthrie (393–394) and Fiona Fleck report on efforts made in Mexico to improve services offered to people with diabetes. Catherine Calderwood (395–396) explains how realistic medicine is being used to question variations in health outcomes across Scotland. 

## Bangladesh, Bolivia (Plurinational State of), Ethiopia, Guatemala, Lao People's Democratic Republic, Liberia, Rwanda

### Who operates, where?

Lisa Marie Knowlton et al. (437–444) compile a geospatial analysis of access to basic surgery. 

## Bangladesh, Ghana, India, Malawi, Nepal, Pakistan, United Republic of Tanzania 

### Evidence for neonatal survival 

Claudia Hanson et al. (453–464) analyse data from trials of community-based care provision.

## Bangladesh, Ghana, Kenya, Malawi, Nigeria, Pakistan, Sierra Leone, South Africa, United Republic of Tanzania and Zimbabwe

### Measured and done

Barbara Madaj et al. (445–452) propose indicators for quality of maternal and newborn care.

## China

### When patients sue

Zhan Wang et al. (430–436) explore the use of malpractice litigation records as an indication of problematic quality.

## Ethiopia

### Improving care for mothers and babies

Maureen E Canavan et al. (473–477) study the introduction of quality measures. 

## India

### Providing for women during childbirth

Gaurav Sharma et al. (419–429) observe the care given to women during labour and delivery. 

## Kenya, Malawi, Namibia, Rwanda, Senegal, Uganda and United Republic of Tanzania

### Where do patients do best?

Margaret E Kruk et al. (408–418) document variation in the quality of care provided to mothers and babies.

## Kyrgyzstan 

### How to improve hospital care for children

Marzia Lazzerini et al. (397–407) do a randomized trial of staff supervision after training paediatricians in 20 hospitals. 

## Global

### Why care about quality?

Yoko Akachi & Margaret E Kruk (465–472) argue that patients’ perceptions of care can be used to improve health services.

### From inevitable to preventable

Peter J Pronovost et al. (478–480) present five narratives that impede progress towards safer care. 

